# Impaired Multisensory Integration Predisposes the Elderly People to Fall: A Systematic Review

**DOI:** 10.3389/fnins.2020.00411

**Published:** 2020-04-28

**Authors:** Sulin Zhang, Wenchao Xu, Yuting Zhu, E. Tian, Weijia Kong

**Affiliations:** ^1^Department of Otorhinolaryngology, Union Hospital, Tongji Medical College, Huazhong University of Science and Technology, Wuhan, China; ^2^Institute of Otorhinolaryngology, Union Hospital, Tongji Medical College, Huazhong University of Science and Technology, Wuhan, China; ^3^Key Laboratory of Neurological Disorders of Education Ministry, Tongji Medical College, Huazhong University of Science and Technology, Wuhan, China

**Keywords:** multisensory integration, balance, older adults, systematic review, neurophysiology

## Abstract

**Background:** This systematic review pooled all the latest data and reviewed all the relevant studies to look into the effect of multisensory integration on the balance function in the elderly.

**Methods:** PubMed, Web of Science and Scopus were searched to find eligible studies published prior to May 2019. The studies were limited to those published in Chinese and English language. The quality of the included studies was assessed against the Newcastle-Ottawa Scale or an 11-item checklist, as recommended by Agency for Healthcare Research and Quality (AHRQ). Any disagreement among reviewers was resolved by comparing notes and reaching a consensus.

**Results:** Eight hundred thirty-nine records were identified and 17 of them were included for systematic review. The result supported our assumption that multisensory integration works on balance function in the elderly. All the 17 studies were believed to be of high or moderate quality.

**Conclusions:** The systematic review found that the impairment of multisensory integration could predispose elderly people to fall. Accurate assessment of multisensory integration can help the elderly identify the impaired balance function and minimize the risk of fall. And our results provide a new basis for further understanding of balance maintenance mechanism. Further research is warranted to explore the change in brain areas related to multisensory integration in the elderly.

## Introduction

### Rationales

Epidemiological studies showed that elderly people aged 60 or above are expected to account for 20% of overall population by 2050 (World Health Organization, 2015; United Nations, 2017). Sufficient mobility, i e., the ability to move physically and engage in daily activities (Lowry et al., 2012) is essential for normal living. Such movement and activities requires maintenance of physical balance (Stel et al., 2003). Moreover, each year, ~30% of community-dwelling elderly people aged 65 and above suffer from falls (Blake et al., 1988) and the rate rose to 32–42% in those aged over 70 (Stalenhoef et al., 2002). The rate of fall-related hospitalization in people at the age of 60 and above was somewhere between 1.6 and 8.9 per 10,000 people (World Health Organization, 2007) and fall-related fatalities reached up to 40% of all injury-related deaths (Rubenstein, 2006). These data show that the balance function is closely associated with the quality of life of the elderly. Mechanistically, balance is achieved or maintained by the integrated input of vision, vestibular and somatic sensation into the central nervous system, and the following responses of the musculoskeletal system (Katsarkas, 1994). It has been substantiated that uni-sensory acuity degrades as a result of aging (Chisolm et al., 2003; Stein and Stanford, 2008), and, presumably, such impaired acuity predisposes elderly people to imbalance and falls (Tromp et al., 2001). Furthermore, elderly people tend to suffer from multiple diseases, including physical and mental disorders, that may well-cause imbalance or falls.

Nonetheless, high sensory acuity is not the only prerequisite for maintaining balance. Balance maintenance may also depend on efficient multisensory integration in the brain. Multisensory integration refers to the process by which the nervous system integrates information from different perceiving processes, such as sight (Wassenhove et al., 2005), hearing (Peelle and Sommers, 2015; Morís Fernández et al., 2017), and other somatosensory events (Foxe et al., 2000), into a unified, coherent, and stable single multisensory process (Bolognini et al., 2015; Talsma, 2015). Furthermore, multisensory integration can compensate for the inadequacy of information from a single sense (Schroeder and Foxe, 2005; Diaconescu et al., 2013; Bizley et al., 2016), provide redundant information that brings about faster detection responses (Kinchla, 1974) and help perceive as much as meaningful information in the environment (Spence, 2011). Such integration has been a subject of active studies on human learning (Seitz et al., 2006), memory (Barutchu et al., 2019), and consciousness (Price, 1999). So far, the physiological and pathological roles of multisensory integration have not been fully understood. However, many studies have shown that, with aging, the multisensory integration is progressively impaired. Some studies showed that multisensory integration is enhanced among the elderly compared to young adults (Laurienti et al., 2006; DeLoss et al., 2013; Ramkhalawansingh et al., 2016) while other researches showed the otherwise (Stephen et al., 2010).

### Objectives

Few studies examined the effect of multisensory integration on the balance function of elderly people (Allison et al., 2006; Bronstein, 2016) and so far, no consistent conclusions have been reached yet. In this systematic review, we pooled all the latest data and comprehensively analyzed the relevant studies to further look into the effect of multisensory integration on balance in the elderly.

### Questions to Be Answered

This study tried to answer the following questions: Does multisensory integration work on balance function in elderly people? Which neurophysiological theories or hypotheses can explain their association?

## Methods

### Study Design

Systematic review.

### Participants, Interventions, Comparators

Participants: elderly people. Interventions: normal multisensory integration. Comparators: impaired multisensory integration. Outcome: balance function.

### Protocol of the Systematic Review Protocol

The protocol for this systematic review was registered with the international PROSPERO database and the trial registration number is CRD42019134526. Protocol details are available from http://www.crd.york.ac.uk/PROSPERO/display_record.php?ID=CRD42019134526.

### Search Strategy

This review was conducted in line with the Preferred Reporting Items for Systematic Reviews and Meta-Analyses (PRISMA) statement (Moher et al., 2009) and consisted of a systematic search of the major databases, formulation of inclusion or exclusion criteria, quality assessment, and data extraction. PubMed, Web of Science (WOS), Scopus were searched to identify qualified studies published before May 2019. The studies were limited to those published in the Chinese language and English language. The following terms were employed: “^*^sensory integration” and “balance OR equilibrium” and “older people,” where “^*^” was the wild card. When we searched multisensory integration, we used “‘Sensory integration’ OR ‘multisensory integration’ OR ‘crossmodal integration’ OR ‘cross-modal integration’ OR ‘intersensory integration’ OR ‘multimodal integration’ OR multisensory OR crossmodal OR cross-modal OR ‘crossmodal sensory integration’ OR ‘cross-modal sensory integration’ OR ‘multisensory interaction.”’ When we searched balance, “‘posture control’ OR ‘postural control’ OR ‘gesture control’ OR ‘postural balance’ OR equilibrium OR balance” is our terms. And we used “geriatr^*^ OR aged OR elderly OR gerontol^*^ OR ‘older adults’ OR aging.” The titles and abstracts of retrieved articles were reviewed and assessed independently by two reviewers to identify eligible studies against the inclusion or exclusion criteria and the full text of the eligible articles was retrieved for further analysis. If any disagreement occurred, a consensus was reached by further referring to the inclusion or exclusion criteria.

### Data Sources, Studies Sections, and Data Extraction

This systematic review pooled the most up-to-date data on the effect of multisensory integration on balance in elderly people. A study was included if (1) the target population was elderly people aged 60 and over (United Nations, 2017); (2) the subjects without unisensory abnormality included were elderly people with fall history or the elderly people in whom assessment showed impaired balance function.

Moreover, studies were excluded if: (1) they were case reports, reviews, letters, or editorial comments; (2) relevant data were insufficient or unavailable; (3) they had overlapping data or were animal studies.

Case control studies were assessed in terms of quality against the Newcastle-Ottawa Scale (NOS) (Wells et al., 2012), which consists of nine sub-items under the headings of the overall selection of groups, the comparability of study groups and the overall ascertainment of the exposure in all groups. For each sub-item, one point was awarded. So, the highest score was nine. In line with other systematic reviews (Ma et al., 2016; de Dieuleveult et al., 2017), five points were selected as cut-off values. Studies that scored five or higher were deemed of high quality. The quality of cross-sectional studies was assessed using an 11-item checklist which was recommended by Agency for Healthcare Research and Quality (AHRQ). An item would be rated “0” if its answer was “NO” or “UNCLEAR;” if the answer was “YES,” “1” point was awarded. Study quality was assessed on an 0–11 scale: low quality = 0–3, moderate quality = 4–7, high quality = 8–11. Two investigators independently assessed the quality of each eligible study. Any disagreement was resolved by reaching a consensus.

By examining the abstract, introduction, methods, and results of each individual study, the features of the studies were identified and listed in a table, including sample size, age range, gender, materials and methods, and results related to the effect of multisensory integration. Data were extracted by using a pre-designed form. Two reviewers separately extracted the data *as per* criteria. Any disagreement was settled by reviewing the full text of the paper involved.

### Data Analysis

The tests used in these studies were too heterogeneous to be pooled. Even though some studies had used the same tests, the test conditions and the measurements varied substantially. In view of these, we only provided a qualitative synthesis of the results.

## Results

**Figure 1:**
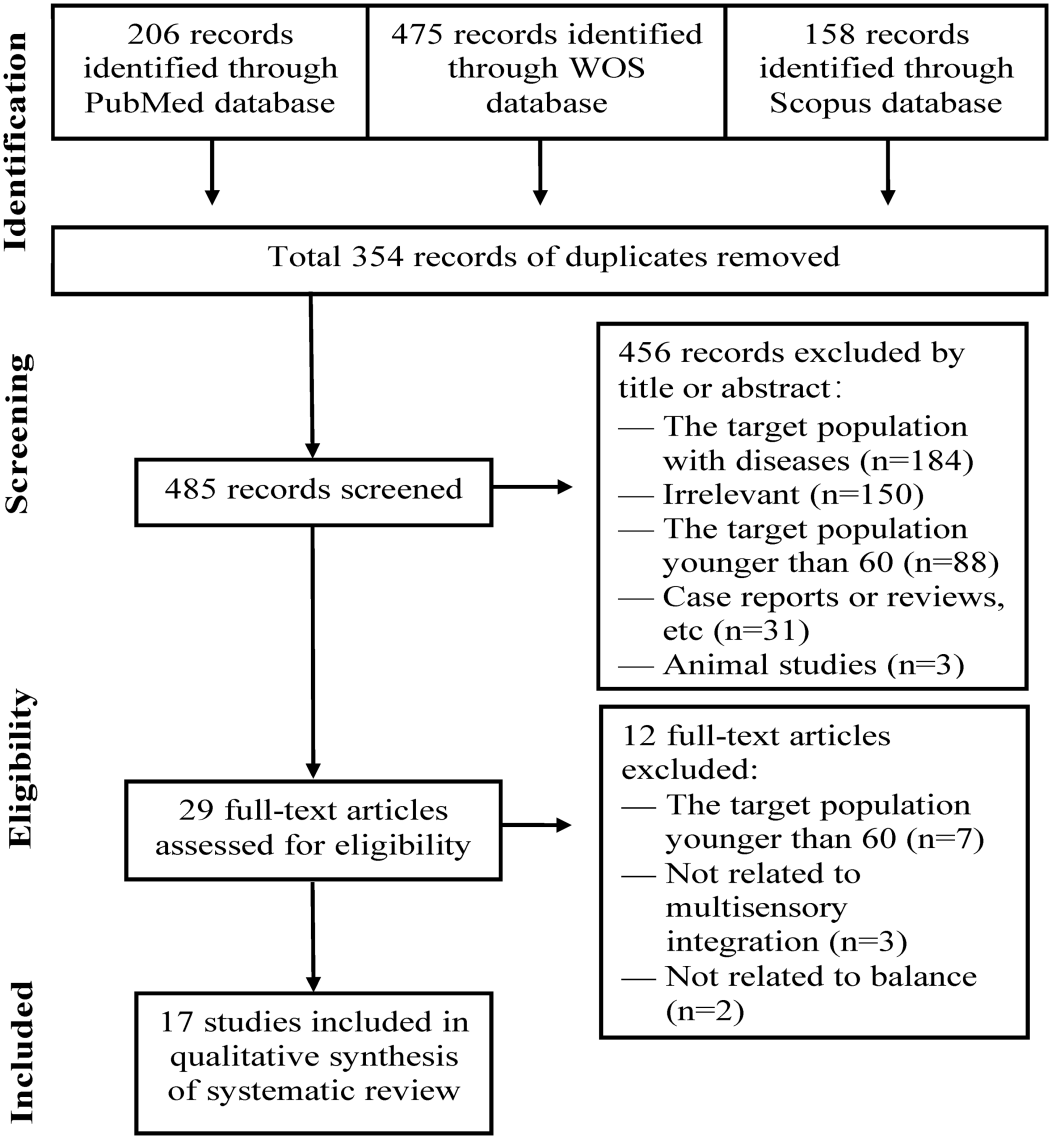
PRISMA flow diagram of study selection process.

The initial database search identified 839 records that were potentially eligible for inclusion in the systematic review. Then, 354 duplicate records were eliminated. Future review of the title and abstract removed 456 records that did not satisfy the inclusion criteria. Upon assessment of the remaining 29 full-text articles, 12 more articles were ruled out. As a result, 17 studies were eventually included for qualitative synthesis of systematic review (Figure 1).

### Study Selection and Characteristics

In these studies, 14 studies were case control studies and three were cross-sectional studies. Against NOS (Table 1), all the 14 studies scored high points on quality, i.e., five or higher and were considered to have strong power. The quality assessment of cross-sectional studies is presented in Table 2. Three studies were of moderate quality.

**Table 1:** Quality assessment with the Newcastle-Ottawa Quality Assessment Scale (NOS).

**References**	**Selection (max** **=** **4)**				**Comparability (max** **=** **2)**		**Exposure (max** **=** **3)**			**Quality score (max = 9)**
	**Is the population definition adequate?**	**Representative-ness of the population**	**Selection of controls**	**Definition of controls**	**Study controls for MSI**	**Study controls for additional factor**	**Ascertainment of exposure**	**Same method of ascertainment for cases and controls**	**Non-response rate**	
Redfern et al. (2001)	^*^	^*^	-	^*^	^*^	^*^	^*^	^*^	^*^	8
Allison et al. (2006)	^*^	^*^	^*^	^*^	^*^	-	^*^	-	^*^	7
Jeka et al. (2006)	^*^	^*^	-	^*^	^*^	-	^*^	^*^	^*^	7
Redfern et al. (2009)	^*^	^*^	-	^*^	^*^	^*^	^*^	^*^	^*^	8
Jeka et al. (2010)	^*^	^*^	-	^*^	^*^	-	^*^	^*^	^*^	7
Setti et al. (2011)	^*^	^*^	^*^	^*^	^*^	^*^	^*^	^*^	^*^	9
Zhou et al. (2013)	^*^	^*^	-	^*^	^*^	-	^*^	^*^	^*^	7
Mahoney et al. (2014)	^*^	^*^	-	^*^	^*^	-	^*^	^*^	^*^	7
Stapleton et al. (2014)	^*^	^*^	-	^*^	^*^	-	^*^	^*^	^*^	7
Ross et al. (2016)	^*^	-	-	-	^*^	-	^*^	^*^	^*^	5
Teramoto et al. (2017)	^*^	^*^	-	^*^	^*^	^*^	^*^	^*^	^*^	8
Mahoney et al. (2018)	^*^	^*^	-	^*^	^*^	^*^	^*^	^*^	^*^	8
Anson et al. (2019)	^*^	^*^	-	^*^	^*^	^*^	^*^	^*^	^*^	8
Redfern et al. (2019)	^*^	^*^	-	^*^	^*^	^*^	^*^	^*^	^*^	8

(^*^), Acceptable; (-), Not acceptable; MSI, multisensory integration.

**Table 2:** Quality assessment of cross-sectional studies.

**Study**	**Rosengren et al. (2007)**	**Palazzo et al. (2015)**	**Sparto et al. (2018)**
**Scores**	6	7	7

In terms of the inclusion or omission of a group of the fall-prone elderly people, all the studies were divided into two groups: studies involving fall-prone elderly people (*n* = 6) and those without including fall-prone elderly people (*n* = 11). The sample size and age range of the 17 studies are summarized in Table 3. In some studies, other factors were also controlled to ensure the comparability between groups, including gender, education background, intelligence quotient (IQ), among others.

**Table 3:** Sample size and age range of the two groups.

**Groups**	**Sample size (range, mean)**		**Age (years)**	
	**Healthy older adults**	**Fall-prone older adults**	**Healthy older adults**	**Fall-prone older adults**
Studies with fall-prone older adults	7–50, 22	15–94, 32	60–93	60–92
Studies without fall-prone older adults	15–376, 103	_	60–88	_

*(-), not reported*.

### Synthesized Findings

The eligible studies were evaluated in terms of participants, study design, and findings related to the effect of multisensory integration. The results of the evaluation are presented in Tables 4, 5.

**Table 4:** Characteristics of studies in the systematic review (*n* = 17).

**References**	**Participants (sample size; age range; mean (SD); percentage of men)**	**Tests**			**Results related to the effect of multisensory integration**
		**Tasks**	**Conditions**	**Instructions**	
**Studies with fall-prone elderly people group (*****n*** **=** **6)**					
Allison et al. (2006)	Healthy older adults: 15; n.r.; 79(3); n.r.; fall-prone older adults: 28; n.r.; 83(4); n.r; young adults: 10; 19–28; n.r. (n.r.); n.r	Postural task	Moving visual stimulus projected onto a screen in a moving room. Participants held their fingertip touching a moving touch plate. Touch amplitude (mm): visual amplitude (mm): (A) 8:2, (B) 4:2, (C) 2:2, (D) 2:4, (E) 2:8	Maintain posture	“Our results did not support the assumption that the multisensory reweighting adaptation process is deficient in healthy and fall-prone older adults, given sufficiently intact peripheral sensation.”
Jeka et al. (2006)	Healthy older adults: 7; 79–84; 81.1(2.12); n.r; fall-prone older adults: 15; 68–84; 80.7(5.47); n.r; young adults: 12; 18–27; 22.0(3.12); n.r	Postural task	(A) Oscillating at different amplitudes; (B) simultaneously oscillating at a single amplitude and translating to the right at different speeds: 4 mm-4 cm/s, 4 mm-1 cm/s, 4 mm-0 cm/s, 8 mm-0 cm/s.	Maintain posture	The four underlyingmeasures considered together showed a highly significant Condition effect (*P* < 0.0001), a marginally significant Group effect (*P* = 0.052), and a marginally significant Group^*^Condition interaction (*P* = 0.094).
Jeka et al. (2010)	Healthy older adullts: 25; 70–93; 76.6(5.6);44%; fall-prone older adults: 17; 72–92; 79.9(7.3); 40%; young adults: 21; 20–30; 23(2.2); 34%	Postural task	The virtual visual scene oscillated sinusoidally 0.4 Hz. The initial amplitude was either 3 or 12 mm. After 60 s the oscillation amplitude switched from 3 to 12 mm or vice versa, and remained at this amplitude for 120 s	Maintain posture	“For fall-prone adults, gains continued to change over the duration of all time segments, demonstrating relatively slow adaptation and implying that the visual reweighting process in fall-prone adults is not fully achieved during the initial change in gain. Healthy older adults showed the fewest long-term changes in gain, suggesting that their initial response was often the most appropriate of the three groups.”
Setti et al. (2011)	Healthy older adults: 16; n.r.; n.r; 56.3%; fall-prone older adults: 16; n.r.; n.r; 25%; young adults: 16; n.r.; 24.4(4); 43.8%	Sound-induced flash illusion	(A) Visual: 1 or 2 flashes, (B) Auditory 2 beeps, (C) Audiovisual: (1) Congruent: 1 flash/1 beep or 2 flashes/2 beeps, (2) Illusory: Onset of beep precede or follow flash, Different SOA	Report the number of flashes, if no flashes, report the number of beeps	“Importantly, the number of illusions experienced by fall-prone older adults was greater than for healthy older and young adults, and the number of illusions they experienced was unaffected by the onset delay between the auditory beeps from 70 to 270 ms.”
Zhou et al. (2013)	Healthy older adults: 50; n.r; 79.55(5.73); 48%; fall-prone older adults: 94; n.r; 81.84(4.69); 50%	Balance test	Standing on (A) a firm surface with eyes open, (B) a firm surface with eyes closed, (C) a foam surface with eyes open, (D) a foam surface with eyes closed	Maintain balance	“The two groups of subjects had a statistically significant difference (*P* < 0.05), except standing on a firm surface with eyes open and closed.”
Stapleton et al. (2014)	Healthy older adults: 21; n.r; 72.2(4.69); 57.1%; fall-prone older adults: 23; n.r; 73.95(4.94); 52.2%	(A) Postural task, (B) Sound-induced flash illusion	(A) Postural position: (1)sitting, (2) standing; (B) Audiovisual: (1) Congruent: 1 flash/1 beep or 2 flashes/2 beeps, (2) Illusory: 1 flash/2 beeps onset of beep precede or lag flash, Different SOA	(A) Maintain posture; (B) report the number of flashes	“There was greater body sway for fall-prone older adults than healthy older adults in both mediolateral and anterior–posterior directional planes. Also, postural sway increased from the presentation of the audio–visual congruent to the audio–visual illusory conditions for the fall-prone older adults only.”
**Studies without fall-prone elderly people group (*****n*** **=** **11)**					
Redfern et al. (2001)	Healthy older adults: 18; 70–85; 74(3.2); 44.4%; young adults: 18; n.r; 22.8(1.8); 55.6%	Postural task	(A) Postural task: (1) seated, (2) fixed floor with a stable visual environment, (3) sway-referenced floor with a fixed visual scene, (4) sway-referenced floor and sway-referenced visual scene; (B) information processing task: (1) none, (2) visual simple reaction time task, (3) an auditory SRT task, (4) an inhibition reaction time task.	(A) Maintain posture, (B) respond as quickly as possible to the stimulus	“However, older subjects' performance of a concurrent information processing task was associated with increased postural sway. This increase in sway in older adults was particularly evident when both the floor and visual scene were sway referenced, which created a high degree of sensory conflict. As postural challenge was increased, RT's increased for both young and older subjects.”
Rosengren et al. (2007)	Healthy older adults: 20; 60–73; 65.2; 0%	Balance test	(A) Normal vision, fixed support; (B) eyes closed, fixed support; (C) vision swayreferenced, fixed support; (D) normal vision, support sway-referenced; (E) eyes closed, support surface sway-referenced; (F) vision and support surface both sway-referenced.	Maintain balance	“A significant main effect for condition [F _(5, 95)_ = 170, *p* < 0.001] was obtained. *Post-hoc* analyses using Tukey HSD procedures revealed that performance on condition 1 was significantly better than that obtained in all of the other conditions. These findings reflect typical of performance on CDP.”
Redfern et al. (2009)	Healthy older adults: 24; 70–82; 74.2(4.4); 50%; young adults: 24; 21–34; 25.7(3.8); 45.8%	(A) Postural task, (B) Inhibition task	(A) Postural task: (1) Visual conditions: (a) Eyes open in the light, (b) Eyes open in the dark, (c) Sway-referenced visual scene; (2) Platform conditions: (a) Fixed support surface, (b) Sway-referenced floor; (B) Inhibition task: (1) Perceptual task: (a) Congruent side arrow pointed and position arrow, (b) Incongruent; (2) Motor task	(A) Maintain posture, (B) (1) Press a button on the side an arrow pointed; (2) Press the button on the side toward the arrow pointed or on the side opposite the arrow pointed	“In the older adults, perceptual inhibition was positively correlated with sway amplitude on a sway-referenced floor and with a fixed visual scene (*r* = 0.68, *p* < 0.001). Motor inhibition was not correlated with sway on either group. Perceptual inhibition may be a component of the sensory integration process important for maintaining balance in older adults.”
Mahoney et al. (2014)	Healthy older adults: 70; n.r; 75(6.09); 42.9%	(A) RT paradigm, (B) Balance test	(A) Two uni-sensory (visual and somatosensory) and one multisensory (simultaneous VS), (B) with one foot on the ground	(A) respond to all stimuli by pressing a stationary foot pedal, (B) Maintain balance	“A one-way ANOVA revealed significant differences in mean unipedal stance time between MSI classification [NO MSI vs. MSI; *F* (1, 69) = 9.51, *p* < 0.01].”
Palazzo et al. (2015)	Healthy older adults: 40; n.r; 70.18(4.28); 42.5%	Balance test	bipedal stance on three different surfaces, in two different visual conditions for each surface: open and closed eyes.	Maintain balance	“The results of this study showed the importance of multisensory stimulation in postural control and in the maintenance of body balance in the orthostatic position which in turn reduced the accident risk such as falls in the elderly.”
Ross et al. (2016)	Healthy older adults: 15; n.r; 78.67(7.73); n.r; young adults: 15; n.r; 19.87(2.10); n.r	Postural task	(A) Eyes closed during silence, (B)eyes open during silence, (C)eyes closed during noise, (D)eyes open during noise	Maintain posture	“Standard deviation in the A-P and M-L sway and radial sway was compared across condition and using two-way analyses of variance (eyes closed vs. open and silence vs. noise). We found main effects ofvision [F _(1, 28)_ = 9.36, *p* = 0.005] and noise [F _(1, 28_) = 5.93, *p* = 0.022] on A-P sway, a main effect of noise [F _(1, 28)_ = 8.86, *p* = 0.006) on M-L sway, and main effects of vision [F _(1, 28_) = 10.47, *p* = 0.003]and noise [F _(1, 28)_ = 9.01, *p* = 0.006] on radial sway.”
Teramoto et al. (2017)	Healthy older adults: 20; 71–82; 74.6(2.9); n.r; young adults: 11; 20–22; 21.4(0.70); n.r	(A) Sensorimotor function assessment, (B) RT paradigm	(A) Timed Up and Go (TUG) and postural stability tests, (B) tactile only, visual only (Vnear, Vmiddle and Vfar), visuotactile (VTnear, VTmiddle, and VTfar)	(A) maintain posture, (B) speeded responses to all stimuli	“The detailed analysis using the TUG and postural stability test scores in the older adults further demonstrated that the enhanced visuotactile interactions were especially prevalent among the older adults with relatively poor TUG and postural stability per formance.”
Mahoney et al. (2018)	Healthy older adults: 289; n.r; 76.67(6.37); 47%	(A) RT paradigm, (B) Balance test	(A) Two unisensory (visual and somatosensory) and one multisensory (simultaneous VS), (B) with one foot on the ground	(A) respond to all stimuli by pressing a stationary foot pedal, (B) Maintain balance	“Maximal unipedal stance time was highest for superior and good integrators (16.43 and 16.83 s) and lowest for poor and deficient integrators (13.49 and 12.57 s). Results from the linear regression analyses reveal that vs. integration is associated with maximum unipedal stance time (β = 0.15, *p* ≤ 0.013).”
Sparto et al. (2018)	Healthy older adults: 222; n.r; 85(3); 45%	Postural task	(A) Stable surface with eyes open or closed, (B) compliant surface with eyes open or closed on	Maintain posture	“In older adults with an average age of 85 years, the control of lateral sway in both quiet standing and a postural tracking task was found to be related to timed chair standing performance and cognitive processing speed, respectively.”
Anson et al. (2019)	Healthy older adults: 376; n.r; 60 and over; n.r; 21 aged 50–60; 17 aged 40–50; 13 adults under age 40	Balance test	(A) Floor with eyes open, (B) floor with eyes closed, (C) foam cushion with eyes open (D) foam cushion with eyes closed	(A) Maintain balance, (B) perceived postural stability	“Overall, sway area increased significantly [*F*_(3, 1690)_ = 302.9, *p* < 0.001] across conditions as the balance tasks became progressively more difficult, and all pairwise comparisons were significant (*p*'s < 0.004).”
Redfern et al. (2019)	Healthy older adults: 34; n.r; 76.0(4.0); 38.2%; young adults: 44; n.r; 23.5(2.9); 27.3%	(A) Postural task, (B) Cognitive testing	(A) Postural task: (1) Visual conditions: (a) fixed visual scene, (b) Eyes closed, (c) Sway-referenced visual scene; (2) Platform conditions: (a) Fixed platform, (b) Sway-referenced platform; (B) Cognitive testing: (1) Perceptual task: (a) Congruent side arrow pointed and position arrow, (b) Incongruent; (2) Motor task	(A) Maintain posture, (B) (1)Press a button on the side an arrow pointed; (2) Press the button on the side toward the arrow pointed or on the side opposite the arrow pointed	“The EQ scores varied across the SOT conditions[*F*_(5, 385)_ = 375, *p* < 0.0001]. There were significant correlations for the older subjects between the EQ scores and four cognitive measures within the SOT conditions.”

*n.r., not reported*.

**Table 5:** Studies grouped in terms of tests used.

**Tests**	**Studies**	**Measurements**	**Differences**
RT paradigm	Mahoney et al. (2014)	Reaction time	Uni-pedal stance time in different multisensory integration
	Teramoto et al. (2017)		Average reaction time based on the TUG scores or postural stability test scores
	Mahoney et al. (2018)		Uni-pedal stance time in different multisensory integration
Sound-induced flash illusion	Setti et al. (2011), Stapleton et al. (2014)	Percentage of correct responses	-
Sensory reweighting	Allison et al. (2006)	Gain and phase	Moving visual stimuli in a moving room and touching a moving touch plate
	Jeka et al. (2006)		Visual display oscillating at different amplitudes
	Jeka et al. (2010)		
Modified clinical test of sensory integration for balance	Rosengren et al. (2007)	Equilibrium scores	Different measurement
	Zhou et al. (2013)	Functional gait assessment scores	Different measurement
	Palazzo et al. (2015)	Center of pressure	Various surfaces with eyes open or closed
	Ross et al. (2016)		A force platform with eyes open or closed under quiet or noisy condition
	Sparto et al. (2018)		Various surfaces with eyes open or closed
	Anson et al. (2019)	Center of mass sway area	Different measurement
Inhibitory function testing	Redfern et al. (2001)	Inhibitory measures	With eyes open or closed
	Redfern et al. (2009)		In the light or in the dark
	Redfern et al. (2019)		With eyes open or closed

### Studies Containing Fall-Prone Elderly People

Studies in this group examined the sway variability when subjects performed experimental tasks in various multisensory integration situations. Participants were required to maintain posture whenever possible in different test situations. First of all, in some studies participants were asked to perform a sound-induced flash illusion task and were exposed to consistent or inconsistent audio-visual stimuli. The subjects were required to record the number of visual or auditory stimuli (Setti et al., 2011; Stapleton et al., 2014). Allison et al. (2006) investigated sway variability of subjects who were asked to look at moving visual stimuli in a moving room and to touch a moving touch plate. Other researchers used the visual display oscillating at different amplitudes, changing from high to low or from low to high amplitudes (Jeka et al., 2006, 2010). And Zhou et al. (2013) employed modified clinical tests for multisensory integration, with subjects standing on a firm or foam surface with eyes open or closed.

All these studies yielded interesting findings. First of all, Setti et al. (2011) and Stapleton et al. (2014) suggested that fall-prone elderly people experienced greater number of illusions than their healthy counterparts, especially under incongruent condition. Inefficient audiovisual integration might predispose the fall-prone elderly people to illusion. So, balance maintenance and the related incidence of falls are believed to be associated with a general impairment in the multisensory integration.

Second, Jeka et al. (2006, 2010) illustrated, in two studies, that poor balance control in the fall-prone elderly people was related to their inability to properly re-weight multisensory inputs. The ability to re-weight sensory inputs is important for postural control in elderly people. Central sensory re-weighting processes are believed to deteriorate with age and are inefficient in fall-prone adults. However, another study, by Allison et al. (2006), didn't support the hypothesis that the multisensory reweighting process is impaired in fall-prone elderly people compared to their healthy counterparts. Nonetheless, they found that postural variability tended to increase when sensory stimulus amplitude or moving speeds increased. The discrepancy among the three studies might be ascribed to the difference in their trial duration, because longer time was needed to reflect actual re-weighting impairment.

Finally, by using a simple technique, Zhou et al. (2013) demonstrated that fall-prone elderly adults tended to have a significantly shorter stance time in modified clinical balance tests for multisensory integration, when standing on a firm or foam surface with eyes open or closed.

### Studies Containing no Fall-Prone Elderly People

Studies in this group investigated participants' ability to maintain balance. Participants were required to achieve balance whenever possible. Some authors examined the postural sway when participants performed modified clinical tests, standing on various surfaces with eyes open or closed (Redfern et al., 2001, 2019; Rosengren et al., 2007; Palazzo et al., 2015; Sparto et al., 2018; Anson et al., 2019) in the light or in the dark (Redfern et al., 2009). Similarly, in some studies, participants were asked to stand on a force platform with eyes open or closed under quiet or noisy condition (Ross et al., 2016). Moreover, subjects were examined for reaction time when they were instructed to quickly react to all stimuli given on one hemispace (left or right), regardless of spatial distance (near/middle/far), and target modality (visual/tactile/visuotactile). And to assess the sensorimotor function of participants, postural stability tests, and the Timed Up and Go (TUG) were used (Teramoto et al., 2017). Some researchers recorded uni-pedal stance time of subjects with different MSI (multisensory integration) (Mahoney et al., 2014, 2018).

Of the 11 eligible studies, eight comparable researches yielded similar results. They found that postural sway increased significantly when balance tasks became progressively more complex, suggesting that the multisensory integration was important in the balance maintenance, and postural control in the upright position. Normal multisensory integration was found to decrease the balance-related incidents, such as falls in elderly people. In three of the eight studies, subjects were asked to perform inhibition tasks and their results revealed that perceptual inhibition might be a component of multisensory integration process (Redfern et al., 2001, 2009, 2019). The ability to isolate and process appropriate sensory stimuli whilst inhibiting irrelevant stimuli is essential for achieving behavioral goals. Teramoto et al. (2017) illustrated that multisensory integration was enhanced, especially in practically all elderly people with poor postural stability. Another two studies (Mahoney et al., 2014, 2018) found significant difference in uni-pedal stance time among subjects with different MSI. Excellent and good integrators had the longest uni-pedal stance time while poor and deficient integrators had the shortest one.

## Discussion

### Main Findings

To understand the effect of multisensory integration on balance function in elderly people, in this study, we searched major databases for eligible articles, conducted a systematic review and found that the multisensory integration and balance were intimately related in elderly people.

**Figure 2:**
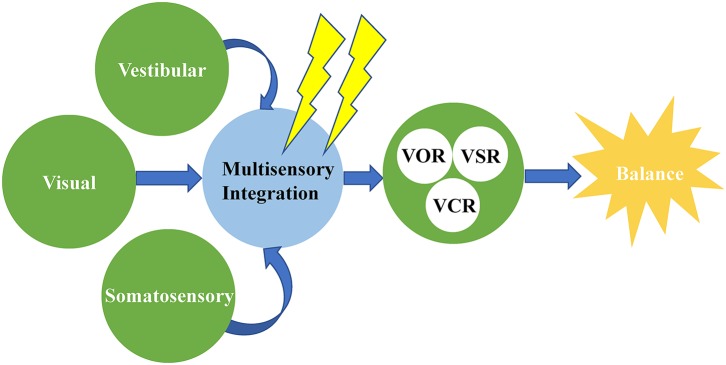
Multisensory integration disorder leads to a decline in balance function in older adults. VOR, vestibulo-ocular reflex; VSR, vestibulospinal reflex; VCR, vestibulocolic reflex.

Balance in elderly adults is multifaceted and is maintained by the integration of vision, vestibular sensation, and somatic sensation inputs into the central nervous system (CNS), and ensuing responses of the musculoskeletal system (Katsarkas, 1994). Any change in these signal inputs can lead to impairment of balance function. Normally, these functions gradually deteriorate with age (Teasdale et al., 1991). Though previously a great many studies looked into the vestibular dysfunction since it is highly prevalent, few researchers examined the balance in the healthy elderly people (Bronstein, 2016). Therefore, in this study, we focused on the balance function in healthy elderly people, with an attempt to understand the effect of multisensory integration on the function. Upon literature searching, we identified 17 papers on the relationship between multisensory integration and balance function in elderly people, and these studies preliminarily suggested that multisensory integration disorder leads to a decline in balance function in elderly people (Figure 2).

Multisensory integration disorder refers to the abnormality in the integration of different sensations or modalities when relevant factors of balance are significantly altered. Impaired information processing in an aging brain has been attributed to prefrontal cortico-cortico facilitation (Knight et al., 1999), dedifferentiation (Dustman and Snyder, 1981), and prefronto-thalamo-cortical gating (Zikopoulos and Barbas, 2006). The neural mechanisms might lie in that peripheral stimuli are analyzed and processed by corresponding sensory cortices, from which afferents are directly transmitted into cortical association area. Cortical association area is one of brain areas where multisensory neurons lie. Also, multisensory neurons have recently been discovered in modality-specific areas, such as the visual cortex, and the junctional zone between cerebral lobes, such as the occipito-temporal space and the occipito-parietal space (Wallace, 2004; Wallace et al., 2004). The subcortical areas of superior colliculus (Wallace, 2004) and putamen (Graziano and Gross, 1993; Gentile et al., 2011) were also found to be involved in multisensory integration. And other researchers believe that the basic structure of sensory system involves a series of neuronal interactions between the thalamus and the neocortex that allow efficient processing of sensory and cognitive information (Sherman and Guillery, 1996; Sherman, 2005). The thalamus plays a vital role in the cortico-cortical communication and the integration of multisensory information. Therefore, damage to cortical areas, subcortical areas, and thalamus may result in the deficits of multisensory integration.

A variety of experimental paradigms have been used to investigate multisensory integration. Of them, illusions, such as ventriloquist illusion (Alais and Burr, 2004; Radeau and Bertelson, 2018), McGurk effect (McGurk and Macdonald, 1976), and sound-induced flash illusion (Shams et al., 2000) are of particular interest to researchers since they help them gain insight into the mechanisms about the management of conflicting multisensory information. The results of this review indicated that elderly people with poor stability tend to integrate or include *all* the information from surrounding environment while elderly people with good stability usually weigh and screen the information to achieve balance. On the basis of the systematic analysis of relevant papers, we reviewed the theories and hypotheses on how multisensory integration works on balance function in elderly people.

### Inverse Effectiveness

First of all, “inverse effectiveness” theory, initially proposed by Meredith and Stein (1986), might, to some degrees, explain how multisensory integration works on balance in elderly people. “Inverse effectiveness” theory believes that the effectiveness of multisensory integration increases when the effectiveness of the best modality-specific stimulus declines (Stein and Stanford, 2008). Some studies suggested that when stimuli from different modalities are congruent, then the benefit of multisensory inputs to perception is greater in elderly than in younger adults (Laurienti et al., 2006; Peiffer et al., 2007). However, when incongruent inputs from different modalities are combined, the combination can result in inefficient processing of the inputs in elderly people (Poliakoff et al., 2006). Given the functional deterioration of sensory system in the eldely, such increased effectiveness of multisensory integration might help the elderly avail of more information from the environment to maintain balance. In the included studies, all participants underwent an intensive clinical assessment, including sensory acuity test, and were found to have no unisensory impairment. These studies showed that even when baseline values were not significantly different, elderly people with poor balance function had less multisensory enhancement (Mahoney et al., 2014). Moreover, the difference between two groups lies in multisensory rather than unisensory processing, suggesting that the difference in the processing of information is of central instead of peripheral nature. It is possible, therefore, that the inverse effectiveness of the balance-related sensations like vision, vestibular sensation and somatic sensation in healthy elderly people is super-additive or additive while fall-prone elderly people only have a sub-additive enhancement (Stein et al., 2009).

### Deficits in Attentional Control

Another theory is “deficits in attentional control.” The attention involves a mechanism that determines how to select sensory inputs for further processing from a series of concurrent stimuli (Talsma et al., 2010). We live in a multisensory world in which we are continuously exposed to stimuli via multiple sensory pathways. For effective cognition, we must continually select and appropriately integrate those inputs that are most relevant to our behavioral goals. Recently, multiple researches suggested that many falls in balance-impaired elderly people occurred not when they were simply walking, but when they were walking and simultaneously performing another task, such as talking (Shumway-Cook and Woollacott, 2000; Woollacott and Shumway-Cook, 2002). These studies supported the notion that attentional demand associated with postural control is higher in fall-prone elderly people. Latinus et al. (2010) believe that stimulus-driven, bottom-up mode induced by integration of multisensory inputs can automatically capture attention toward multisensory events. Conversely, with the top-down mode, attention can facilitate the integration of multisensory inputs and lead to a spread of attention across sensory modalities. Recent studies showed that multisensory bottom-up processes are conductive to the capture and selection of attention. In turn, attention can affect the effectiveness of multisensory integration in a top-down fashion. Andres et al. (2006) demonstrated that the elderly had deficits in attentional control and were more likely to be distracted by stimuli from different sensory modalities. These findings suggest that there exists a closer and multifactorial interaction between attention and multisensory integration. The dynamic and bidirectional interplay between attentional selection and multisensory processing is fundamental to postural control and balance maintenance. Therefore, when the elderly try to maintain balance and simultaneously perform another task, the activity of the brain region associated with balance is significantly reduced while the brain areas of unrelated modalities are, on the contrary, more active (Mozolic et al., 2012), especially in fall-prone elderly people. The beneficial effect of attentional control on balance maintenance are reduced and fall results.

### Larger Time Window of Integration

Finally, another important theory concerning multisensory integration is the “larger time window of integration” hypothesis. A common finding across many studies was that elderly people had an increased response time (Diederich et al., 2008; Mozolic et al., 2012). Diederich et al. (2008) used a time-window-of-integration (TWIN) model to distinguish between the relative contributions of early peripheral sensory processes and subsequent central integration to multisensory enhancement. They found that the larger time window of integration in the elderly is primarily the result of slower and more variable peripheral sensory processing. Sound-induced flash illusion is one of the most common phenomena resulting from larger time window of integration. When auditory and visual stimuli are presented rapidly, the number of auditory stimuli can affect the number of visual stimuli perceived (Bizley et al., 2016). Studies on the sound-induced flash illusion showed that the integrated time window was larger in fall-prone elderly people (Setti et al., 2011; Stapleton et al., 2014). Bloem et al. (2006) found evidence for a “posture-first” strategy, by which participants performing dual-tasks sacrificed performance on perceptual tasks and prioritized balance control. Therefore, the fall-prone elderly people have less allocation of attentional resources to the multisensory task when maintaining balance. Such insufficient allocation leads to an increase in faulty percepts when multisensory information is incongruent (Stapleton et al., 2014). But how exactly the larger time window of integration affects balance is still unknown. Setti et al. (2011) put forward a speculation that either an indirect or a direct effect might be involved in the mechanism. On the one hand, balance can be indirectly challenged by the processing of irrelevant sensory information due to the larger time window. On the other hand, fall-prone elderly people tend to over-depend on multisensory stimulation. Therefore, when exposed to incongruent stimuli, fall-prone elderly people have a specific difficulty in processing the information. In turn, the larger temporal window may exert a direct effect on their ability to maintain balance.

Some other theories should also be mentioned here. It has been shown that the brain of older adults tends to have less asymmetric hemispheric activation, a phenomenon called HAROLD (hemispheric asymmetry reduction in older adults) (Cabeza, 2002). And it states that, during multisensory tasks, the elderly recruit more brain areas. HAROLD was found to be correlated with higher performance in task execution in the elderly, which prompts a hypothesis that these changes take place to preserve cognitive function in elderly people. One possible explanation is that the mechanism is a compensation for aging (Peters, 2006). However, the relationship between brain recruitment strategies and balance maintenance has not yet been fully understood. Another explanation concerns the increased noise at baseline (Mozolic et al., 2012). When elderly people are engaged in selective attention, multisensory enhancement in their brain remains and can benefit them when all information is reliable. However, when some irrelevant information is present, this increased baseline becomes a disadvantage.

The major finding of the study was that appropriate integration of information from multisensory modalities is essential for elderly people to maintain balance. The hypothesis of deficits in attentional control and larger time window of integration can better explain why the multisensory integration impairment is associated with impaired balance function in elderly people.

### Strengths and Limitations

Our review has several strengths. This systematic review was based on the guidelines of the PRISMA statement (Moher et al., 2009), a tool designed to enhance the quality of systematic reviews. All accessible databases were searched, including WOS, PubMed, Scopus, among others, which maximized the number of eligible studies. What's more, the quality of case control studies was assessed against NOS (Wells et al., 2012). Only high-quality studies were included in qualitative synthesis. The quality of cross-sectional studies was assessed using an 11-item checklist which was recommended by AHRQ. Most importantly, our systematic review found that elderly people with poor stability tend to integrate or include *all* the information from surrounding environment while elderly people with good stability usually weigh and screen the information to achieve balance. Moreover, multisensory integration might work on balance function in elderly people.

This study had some limitations. The eligible studies yielded different results concerning the effect of multisensory integration, so it was impossible to conduct a meta-analysis. And the different methods used in the 17 included studies also made it difficult to analyze the overall effect of the multisensory integration on balance function. Furthermore, we only reviewed the abstracts and full texts that were published in English and Chinese. Relevant papers in other languages might be not included. And some relevant studies might be missed if authors didn't list their studies as being related to multisensory integration.

## Conclusions

In conclusion, this systematic review looked into how multisensory integration works on balance function in elderly people and found that the impairment of multisensory integration will predispose elderly people to fall. Accurate assessment of multisensory integration can help the elderly identify the impairment of balance function and minimize the risk of fall. And our results provide a new basis for further understanding of mechanisms of balance maintenance. Further research is warranted to explore the changes in brain areas related to multisensory integration in elderly people.

## Data Availability Statement

The raw data supporting the conclusions of this article will be made available by the authors, without undue reservation, to any qualified researcher.

## Author Contributions

SZ and WX: study conception, design, data extraction, data analysis and interpretation, and drafting and revision of the manuscript. YZ and ET: data extraction and critical revision. WK: study design and critical revision. All authors approved the manuscript.

## Conflict of Interest

The authors declare that the research was conducted in the absence of any commercial or financial relationships that could be construed as a potential conflict of interest.
